# Tropical peatland microbiomes as a One Health nexus: linking soil, plant, and climate health across land-use transitions

**DOI:** 10.3389/fmicb.2026.1931180

**Published:** 2026-10-27

**Authors:** Selva Dhandapani

**Affiliations:** Soil Biogeochemistry and Terrestrial Ecology Research Programme, Agri-Food and Biosciences Institute, Belfast, United Kingdom

**Keywords:** carbon cycling, climate change, ecosystem services, microbiome, oil palm, One Health, peatland policy, restoration

## Abstract

The One Health framework recognizes the intrinsic connections among environmental, plant, animal and human health. While soils and soil microbes have received substantial attention in the recent years, tropical peatlands still remain largely overlooked despite representing globally important reservoirs of carbon, biodiversity, freshwater. Microorganisms are central regulators of peat accumulation, nutrient cycling, greenhouse gas exchange and ecosystem resilience, yet their role as mediators of One Health outcomes has received limited attention. Emerging evidence from tropical peatlands demonstrates that land-use change, drainage, fire and agricultural conversion substantially alter microbial community composition and function, with cascading effects on ecosystem processes. In this Perspective, peatland microbiomes are proposed as critical but neglected One Health nexus linking soil health, vegetation dynamics, wildlife habitat quality, climate regulation and human wellbeing. Considering, oil palm plantations that produce the most consumed vegetable oil in the world, cover substantial portion of tropical peatlands, tropical peat also plays a crucial role in food security and safety. The perspective argues that microbial communities should move from being considered ecological indicators to becoming policy-relevant components of peatland governance. Furthermore, incorporation of microbiome informed indicators into restoration, carbon accounting and ecosystem health frameworks is proposed as a necessity. By integrating microbial ecology with One Health principles, tropical peatlands can serve as an impactful model system for understanding how belowground biodiversity underpins ecosystem services and planetary health.

## Introduction

The One Health concept has evolved from a predominantly human and animal health framework into a broader recognition that environmental integrity underpins the health of all living systems (Jones et al., 2025). Soils occupy a central position in this framework because they regulate nutrient cycling, primary productivity, biodiversity and ecosystem resilience, processes that are primarily mediated by microorganisms (Adhikari and Hartemink, 2016). Soils are the source of all essential nutrients for life, and 80% of antibiotics that form the basis for modern medicine (Han et al., 2022). Soil microbiomes form the basis for nutrient and carbon cycling, and most of the antibiotics are produced from the secondary metabolites of microorganisms such as bacteria and fungi (Sengupta et al., 2013). Considering that microbes form the basis for important services provided by the soil that are essential for life on earth, it is no surprise that soils are home to ∼60% of all biodiversity on earth, most of which are microbes (Anthony et al., 2023).

Microbiome is a term that integrates microbe and biome to describe the entirety of a microbial ecosystem (Berg et al., 2020; Tomasulo et al., 2024). It encompasses not only the community of microorganisms themselves (the microbiota), including bacteria, archaea, fungi, protists and viruses, but also all the molecules they produce, such as nucleic acids, proteins, lipids, polysaccharides and metabolites, as well as molecules generated through interactions with coexisting hosts and shaped by surrounding environmental conditions (Berg et al., 2020). Thus, the microbiome represents both the biological community and its collective functional potential, providing a mechanistic link between biodiversity and ecosystem processes.

Understanding microbiomes is particularly important in unique, extreme, rare and relatively unexplored environments such as tropical peatlands. These ecosystems are characterized by acidic, nutrient-poor, waterlogged and often anoxic conditions that have driven the evolution of highly specialized microbial communities (Too et al., 2018; Dhandapani et al., 2019c). Despite occupying a relatively small proportion of the Earth’s surface, tropical peatlands store globally significant carbon stocks, perhaps the most carbon dense ecosystem in the world, and provide critical ecosystem services, including climate regulation, biodiversity conservation and hydrological regulation (Yule, 2010; Page et al., 2011). Many of these functions are fundamentally controlled by microbial processes governing peat formation, decomposition, nutrient cycling and greenhouse gas exchange. Thus, tropical peatlands represent an exceptional yet underappreciated component of the One Health continuum. Yet, compared with temperate peatlands and agricultural soils, tropical peatland microbiomes remain poorly understood, representing one of the last major frontiers in soil microbial ecology.

Anthropogenic pressures from drainage, fire, agricultural expansion and climate change continue to intensify across tropical peatland regions (Page and Hooijer, 2016; Dhandapani and Evers, 2020; Dhandapani et al., 2023). These disturbances alter environmental conditions that have historically shaped microbial assembly and functioning over millennia (Mishra et al., 2014; Dhandapani et al., 2019a,c; Dhandapani et al., 2020b; Birnbaum et al., 2025). Consequently, microbial responses may provide an early-warning signal of ecosystem degradation and a mechanistic explanation for changes in ecosystem service delivery. This perspective argues that tropical peatland microbiomes should be recognized as a critical One Health nexus connecting soil, plant, animal, human and climate health; and provide a conceptual framework linking microbial community composition with ecosystem services and discuss how microbiome-informed approaches could transform peatland management and policy.

## From invisible biodiversity to ecosystem engineers

Microbial communities are fundamental architects of peatland ecosystems (Andersen et al., 2013). Tropical peat accumulates because decomposition is constrained by acidic, nutrient-poor and waterlogged conditions (Page et al., 2011). Under these conditions, microbial processes regulate the balance between carbon storage and carbon loss, thereby determining whether peatlands function as carbon sinks or greenhouse gas sources (Andersen et al., 2013). Microorganisms are the hidden engineers of peatland ecosystems. Consequently, peatland ecosystem health cannot be fully understood through vegetation, hydrology and biodiversity assessments alone. The ecological functioning of tropical peatlands depends fundamentally upon microbial communities operating throughout the peat profile (Andersen et al., 2013).

Over the last two decades, research on tropical peatland microbiomes has expanded from a handful of descriptive studies to increasingly sophisticated investigations using molecular ecology, metagenomics and ecosystem-scale analyses (Table 1). Collectively, these studies reveal that peatland microbiomes are central regulators of ecosystem functioning and may represent a critical but overlooked component of One Health and Planetary Health frameworks.

**TABLE 1:** Studies exploring tropical peat microbial communities, methods used for microbial characterization, core findings and their implications for planetary and One Health.

References	Method	Findings	Implications for planetary/One Health
Tawaraya et al., 2003	Microscopic assessment of arbuscular mycorrhizal colonization in roots of 22 tropical peat-swamp tree species from Central Kalimantan, Indonesia.	Arbuscular Mycorrhizal (AM) fungi colonized 77% of species surveyed, including several dominant peat-swamp tree species. The authors proposed that AM fungi could improve seedling growth and aid peatland rehabilitation.	Reveals the importance of beneficial soil microbes in supporting forest regeneration, nutrient cycling and restoration of degraded peatlands, with downstream benefits for biodiversity, carbon storage and planetary health.
Turjaman et al., 2005	Peat-grown *Shorea pinanga* seedlings inoculated with ectomycorrhizal fungi (*Pisolithus arhizus* and *Scleroderma sp*.) in a nursery experiment.	Ectomycorrhizal colonization exceeded 86%, significantly improving growth and increasing seedling survival from 16% to 73%–91%.	Highlights the importance of beneficial soil microbes for peatland forest restoration, biodiversity recovery and long-term carbon storage, supporting One Health and planetary health objectives.
Turjaman et al., 2006	Peat-grown *Shorea seminis* seedlings inoculated with *Pisolithus arhizus* and *Scleroderma columnare*; ECM colonization, growth and nutrient uptake assessed.	Ectomycorrhizal (EM) fungi increased colonization, biomass, shoot growth, and N and P uptake in seedlings. Higher nutrient uptake was positively associated with plant growth.	Shows how beneficial peatland microbes can enhance forest regeneration and nutrient cycling, supporting restoration of degraded peatlands, biodiversity conservation, carbon storage and climate resilience.
Voglmayr and Yule, 2006	Morphological (light microscopy, SEM) and molecular (ITS and LSU rDNA sequencing) characterisation of fungi isolated from peat swamp forest litter.	Discovered and described *Polyancora globosa*, a novel aeroaquatic fungal genus and species adapted to extreme tropical peatland conditions.	Highlights tropical peatlands as reservoirs of unique and poorly understood microbial biodiversity whose conservation is important for ecosystem resilience, biodiversity protection and planetary health.
Yanai et al., 2007	Fungal isolation and 18S rDNA identification of microorganisms involved in N_2_O production from cultivated tropical peat soils.	Fungi contributed disproportionately to N_2_O emissions, with fungal inhibition reducing emissions by 81%. N_2_O producing strains included *Fusarium oxysporum* and *Neocosmospora vasinfecta*.	Reveals that peatland fungi can strongly influence greenhouse gas fluxes and climate impacts, emphasizing the importance of microbiome management for peatland restoration, climate change mitigation.
Jackson et al., 2009	16S rRNA-based microbial community profiling and extracellular enzyme assays across peat depth gradients.	Microbial communities and functions changed strongly with depth; Acidobacteria and Crenarchaeota dominated bacterial and archaeal communities, respectively. Most microbial activity occurred in surface peat layers.	Provides evidence that peatland microbiomes underpin carbon sequestration, nutrient cycling and ecosystem functioning, making them critical components of climate regulation and planetary health.
Kamal et al., 2009	Screening of 85 microbial isolates from Malaysian habitats, including peat soils, for photo-cytotoxic compounds using a photodynamic therapy assay.	Phototrophic bacteria produced bacteriochlorophyll-derived compounds with strong light activated anticancer activity.	Demonstrates that peatland associated microbial biodiversity may provide novel therapeutics, linking ecosystem conservation directly to human health and bioprospecting opportunities.
Etto et al., 2012	SSU rRNA gene sequencing of bacterial and archaeal communities in Brazilian peatlands.	Acidobacteria and Proteobacteria dominated bacterial communities, while Crenarchaeota dominated archaeal communities. Hydrogenotrophic methanogens were the principal methane producers.	Shows that peatland microbiomes drive carbon and methane cycling, influencing greenhouse gas (GHG) emissions, ecosystem resilience and climate regulation.
Troxler et al., 2012	Plant community surveys, bacterial community profiling, enzyme activity assays and soil chemistry analysis along a tropical peatland phosphorus gradient. PCR-DGGE, 16S rRNA sequencing, enzyme assays, microbial respiration.	Plant and bacterial community composition covaried strongly with phosphorus availability. Acidobacteria dominated bacterial assemblages.	Highlights the importance of plant-microbe interactions in regulating peatland nutrient cycling, carbon storage and ecosystem resilience, linking peatland biodiversity directly to climate regulation and planetary health.
Graham et al., 2013	Field test of mycorrhizal-inoculated seedlings across degraded peatland restoration sites.	Inoculated seedlings maintained higher colonization and nutrient uptake following transplantation.	Informs benefits of microbial inoculation for forested peat restoration.
Kwon et al., 2013	Experimental drought incubation of peat soils from pristine and drained tropical peatlands, coupled with microbial and biogeochemical analyses. Terminal restriction fragment length polymorphism (T-RFLP) profiling of bacteria, archaea and fungi; qPCR; extracellular enzyme assays.	Drained peat soils released more CO_2_, CH_4_, and DOC and showed greater shifts in microbial communities than pristine peat soils. Drought altered microbial communities, especially in drained peatlands, leading to higher carbon losses.	Shows that peatland drainage amplifies climate-change vulnerability by enhancing microbial carbon loss during drought, threatening both ecosystem stability and global climate regulation. Hydrological disturbance modifies microbial processes that regulate GHG emissions.
Mishra et al., 2014	16S rRNA T-RFLP, clone sequencing, metabolomics (UPLC-TOF-MS).	Water-table depth and land-use type were major drivers of microbial community structure; mixed-crop systems supported greater diversity than oil-palm monocultures.	Demonstrates how land-use and hydrological change reshape peatland microbiomes and influence ecosystem sustainability.
Arai et al., 2014a	MPN methanotroph counts, pmoA PCR-DGGE, sequence analysis, and methane-incubation assays.	Methanotrophic communities were largely unaffected by drainage and fire, with Methylomonas-like methanotrophs dominating and methane oxidation exceeding production under most conditions.	Highlights the role of resilient methane-oxidizing microbes in mitigating GHG emissions from tropical peatlands and maintaining ecosystem climate regulation functions.
Arai et al., 2014b	Fumigation extraction microbial biomass analysis and MPN assays for nitrifiers and denitrifiers.	Cropland peat soils emitted more CO_2_ and N_2_O than forest soils, with microbial biomass influencing emissions differently across land uses.	Shows that agricultural conversion reshapes microbial processes controlling GHG emissions, increasing the climate impacts of tropical peatlands.
Espenberg et al., 2018	Metagenomics and qPCR targeting microbial communities and nitrogen-cycling genes.	Drainage altered microbial community structure, reduced nitrogen-fixation and denitrification potential, and increased abundance of ammonia oxidizing archaea and N_2_O emissions.	Highlights how peatland drainage disrupts microbial nitrogen cycling, potentially increasing GHG emissions and weakening ecosystem sustainability.
Könönen et al., 2018	Microbial biomass analysis, extracellular enzyme assays, FTIR spectroscopy, and dissolved organic carbon/nitrogen measurements.	Drained and deforested peatlands showed lower microbial biomass, reduced enzyme activity, and poorer peat substrate quality than intact swamp forests; reforestation had not yet restored microbial function.	Highlights how land-use change degrades peatland microbial processes that support carbon storage, ecosystem recovery, and climate regulation.
Kusai et al., 2018	Culture-dependent fungal isolation on PDA and MEA, morphological characterization, 18S rRNA gene amplification and sequencing, phylogenetic analysis, and soil physicochemical measurements.	Fungal communities differed across primary forest, logged-over forest, and oil palm plantations. *Ascomycota* dominated (∼90% of isolates), with *Aspergillus* and *Penicillium* prevalent in forests and *Hypocrea* species common in oil palm plantations. Most fungi were saprophytes involved in peat decomposition.	Demonstrates that land-use change alters peatland fungal community composition, potentially affecting decomposition processes, nutrient cycling, carbon sequestration and long-term ecosystem resilience.
Too et al., 2018	16S rRNA metabarcoding and environmental measurements across tree species and peat depths.	Peat depth significantly shaped microbial diversity and composition; Proteobacteria dominated, nitrogen availability was a key driver, and distinct methane cycling microbes were identified.	Demonstrates how peatland microbial communities regulate carbon, nitrogen, and methane cycling, supporting climate regulation and ecosystem health.
Dhandapani et al., 2019a	PLFA microbial community profiling, GHG measurements, and peat physicochemical analyses.	Continued oil palm monocropping altered microbial communities and degraded peat properties, whereas intercropping maintained higher organic matter, moisture, and ecological stability.	Sustainable intercropping can reduce peatland degradation, helping protect carbon storage, climate regulation, and ecosystem health.
Dhandapani et al., 2019b	PLFA microbial profiling, GHG measurements, and peat physicochemical analyses.	Mature oil palms increased CO_2_ emissions through root respiration, while microbial community structure in surface peat remained largely unaffected by sampling proximity to crops. Gram-positive bacteria dominated across all systems.	Highlights how agricultural management of peatlands influences carbon losses and GHG emissions, informing strategies for climate change mitigation, ecosystem function, and soil health.
Dhandapani et al., 2019c	PLFA microbial community profiling, GHG measurements, and nutrient analyses.	Drainage and logging shifted microbial communities toward Gram-positive bacteria, increased CO_2_ emissions, reduced nutrient concentrations, and transformed the secondary forest into a methane sink.	Shows how peatland disturbance changes microbial ecology and carbon cycling, affecting climate regulation and ecosystem health.
Dhandapani et al., 2020b	PLFA microbial community profiling of peat cores across land-use types and depths (0–200 cm).	Oil palm conversion, drainage, and burning significantly altered microbial community structure. Bacteria dominated all peat layers, with Gram-positive bacteria more prevalent near the surface and Gram-negative bacteria increasing with depth.	Microbial responses to peatland disturbance can be seen across the peat depth and may influence carbon storage supporting the need for sustainable peatland management and restoration.
Goh et al., 2020	Bacterial isolation using an iChip, 16S rRNA gene sequencing, whole-genome sequencing, phylogenetic analysis, and antimicrobial activity screening against human pathogens.	Identified a novel peat-swamp bacterium, *Chitinophaga extrema sp. nov.*, with broad-spectrum antimicrobial activity and distinct genomic and phenotypic characteristics.	Demonstrates that tropical peatlands are reservoirs of previously unknown microbial diversity and potential sources of novel antimicrobial compounds relevant to antimicrobial resistance.
Mahdiyah et al., 2020	Bacterial isolation and culturing from peat soil, phenotypic identification, agar well-diffusion antimicrobial screening, SDS-PAGE protein analysis, electroelution purification, and MIC/MBC testing.	Three peat bacterial isolates produced antimicrobial proteins with activity against ESBL-producing *E. coli* and MRSA, showing bactericidal effects *in vitro*.	Highlights tropical peatlands as potential sources of novel antimicrobial compounds that could help address antimicrobial resistance, an important One Health challenge.
Liu et al., 2020	Pyrosequencing of fungal ITS and bacterial 16S rRNA genes.	Natural peat forests had the highest fungal richness and distinct microbial communities, while disturbed peat soils contained more pathogenic fungi and altered bacterial groups linked to carbon and methane cycling.	Demonstrates that peatland disturbance changes microbial biodiversity and ecosystem functioning, with consequences for carbon storage, GHG emissions, ecosystem health, and climate resilience.
Kusai and Ayob, 2020	Bacterial isolation and 16S rRNA sequencing across peat depth profiles.	Proteobacteria and Firmicutes dominated peat soils, with community composition varying by depth and land use, especially in drained oil palm plantations.	Demonstrates that peatland drainage and agricultural conversion modify microbial communities involved in carbon and nutrient cycling, with implications for ecosystem functioning and climate regulation.
Girkin et al., 2020	PLFA and GDGT lipid biomarker analyses to characterize bacterial, fungal, and archaeal communities in tropical peat soils.	Gram-negative bacteria dominated microbial communities, while moisture, pH, and redox conditions strongly shaped microbial abundance and methane cycling organisms.	Demonstrates that peatland microbial communities regulate carbon and methane cycling, making them critical for climate stability, ecosystem resilience, and Planetary Health.
Morrison et al., 2021	16S rRNA and ITS2 metabarcoding, microbial trait analysis, and network analysis.	Low-phosphorus tropical peat soils contained distinct fungal and bacterial communities, including microbial consortia linked to methane cycling and carbon turnover.	Microbial interactions in nutrient poor peatlands may regulate GHG emissions and carbon storage, highlighting the importance of peatland conservation for climate and ecosystem health.
Buessecker et al., 2021	16S rRNA gene sequencing, mcrA gene sequencing, qPCR of methanogens, and microbial enrichment/incubation experiments.	Nitrogen oxides (NO_2_^–^ and N_2_O) inhibited methanogenesis, while methanogen and nitrogen cycling communities varied across peatland types.	Demonstrates that nitrogen cycling can regulate methane emissions from tropical peatlands, affecting greenhouse gas fluxes and climate feedbacks.
Dom et al., 2021	16S rRNA amplicon sequencing (Illumina MiSeq) of peat soil prokaryotic communities across forest types and depths	Acidobacteria and Proteobacteria dominated peat soils, while microbial diversity and composition varied with forest type, depth, pH, humification, and organic matter content.	Highlights the central role of peatland microbes in methane regulation, organic matter decomposition, and carbon cycling, supporting climate regulation and ecosystem sustainability.
Too et al., 2021	Whole-genome sequencing and Biolog carbon-utilization profiling of peatland bacterial isolates.	Identified bacteria involved in lignocellulose degradation, methane metabolism, and nitrogen transformations including DNRA	Highlights the role of peatland bacteria in carbon storage, methane mitigation, nutrient retention, and ecosystem resilience, supporting climate and environmental health.
Pavia et al., 2023	Genome resolved metagenomics of Amazon peatlands across nutrient and geochemical gradients.	Peatland geochemistry shaped microbial communities and functional genes involved in carbon degradation, methane cycling, nutrient acquisition, denitrification, and sulfur metabolism.	Shows that microbial responses to nutrient and environmental gradients regulate GHG production and carbon storage in Amazon peatlands, with implications for climate resilience and ecosystem sustainability.
Azizan et al., 2024	16S rRNA Illumina sequencing of peat microbial communities across peat forest and oil palm plantation sites.	Land-use change altered the abundance of key microbial taxa linked to carbon, sulfur, and nitrogen cycling, despite similar overall diversity.	Highlights microbial responses to peatland conversion and their importance for carbon storage, GHG regulation, ecosystem resilience, and sustainable peatland management.
Kusai et al., 2025	Culture-based fungal isolation and 18S rDNA sequencing with community diversity analyses.	*Ascomycota* dominated peat ecosystems; fungal diversity was highest in logged-over forest and differed between peat forests and oil palm plantations.	Demonstrates the importance of fungal communities in decomposition, carbon cycling, ecosystem stability, and sustainable peatland management.
Midot et al., 2025	Shotgun metagenomic sequencing, GHG flux measurements (CO_2_, CH_4_, N_2_O), peat physico-chemical analyses, and functional gene profiling of microbial carbon and nitrogen cycling during peat forest conversion to oil palm plantation.	Conversion to oil palm altered peat physico-chemistry, reduced microbial diversity, and shifted microbial community composition and functional potential. Increased CO_2_ and N_2_O emissions were associated with changes in methanogenic and denitrifying communities and microbial carbon and nitrogen cycling pathways.	Demonstrates that land-use change restructures peatland microbial processes controlling GHG emissions, with consequences for climate regulation, ecosystem sustainability, and long-term peatland health.
Zahari et al., 2026	Soil DNA extraction, 16S rRNA amplicon sequencing, ITS2 fungal sequencing, QIIME2 bioinformatics, ASV analysis, and microbial co-occurrence network analysis.	Conversion of tropical peat swamp forest to oil palm plantations altered bacterial and fungal community composition, reduced network connectivity, and shifted microbial interactions, although alpha diversity remained stable.	Demonstrates how land-use change reshapes peatland microbiomes, potentially affecting nutrient cycling, carbon storage, biodiversity conservation, and climate regulation.
Gao et al., 2026	16S rRNA sequencing, qPCR, ecological network analysis, and phosphorus cycling gene prediction in burned and unburned tropical peatlands.	Wildfire reduced bacterial diversity but increased inorganic phosphorus levels through enrichment of opportunistic bacterial taxa and phosphorus-mineralisation genes.	Shows that post-fire microbial reassembly can regulate nutrient availability and ecosystem recovery, highlighting the importance of microbial resilience for peatland restoration and ecosystem health.

The earliest tropical peatland microbiology studies focused primarily on documenting microbial diversity and exploring beneficial microbial interactions. Tawaraya et al. (2003) demonstrated that arbuscular mycorrhizal fungi colonized most tree species examined in Central Kalimantan peat swamp forests, suggesting an important role in nutrient acquisition and forest regeneration. Similarly, Turjaman et al. (2005, 2006) showed that ectomycorrhizal inoculation dramatically improved survival, growth and nutrient uptake of *Shorea* seedlings, providing some of the earliest evidence that manipulation of peatland microbiomes could directly improve restoration outcomes. These studies established that microorganisms are not merely passive inhabitants of peat ecosystems but active contributors to plant health and ecosystem recovery.

At the same time, investigations of peatland fungal diversity revealed the remarkable uniqueness of microbial life within tropical peatlands. Voglmayr and Yule (2006) described *Polyancora globosa*, a novel fungal genus in submerged litter adapted to peat swamp environments, highlighting tropical peatlands as reservoirs of previously unknown microbial biodiversity. Such discoveries suggest that peatlands may contain vast numbers of undescribed microorganisms with ecological, evolutionary and potentially biotechnological significance.

The importance of these microorganisms extends beyond ecosystem functioning. Kamal et al. (2009) demonstrated that microorganisms isolated from Malaysian habitats, including peat soils, produced photoactive compounds with potential anticancer applications. Goh et al. (2020), Mahdiyah et al. (2020) found microbes with broad-spectrum antimicrobial activities. These finding introduces an additional dimension of ecosystem value: tropical peatlands may serve as reservoirs of microbial genetic resources with direct relevance to human health.

## Tropical peatland microbiomes as regulators of ecosystem health

Jackson et al. (2009) provided the first detailed evidence that tropical peatland microbiomes exhibit strong vertical stratification, with distinct microbial communities occupying different depths. Acidobacteria, Crenarchaeota and other microbial groups were distributed according to gradients in oxygen availability, substrate quality and nutrient resources. Extracellular enzyme activity was concentrated in surface peat, demonstrating that microbial metabolism varies substantially throughout the peat profile. Subsequent studies reinforced this view. Etto et al. (2012) identified distinct bacterial and archaeal assemblages dominated by Acidobacteria, Proteobacteria, and hydrogenotrophic methanogens, highlighting microbial control over methane production and carbon cycling. Troxler et al. (2012) further demonstrated strong coupling between vegetation structure and soil microbial communities along nutrient gradients, revealing important plant-peat-microbe feedback mechanisms regulating peat development and carbon accumulation. Such plant-peat feedback underpin peatland ecosystem services that are critical for climate stability, biodiversity conservation and planetary health.

Recent investigations have revealed highly diverse microbial communities in tropical peat swamp forests, with strong stratification across depth profiles. Microbial diversity tends to decline with increasing depth, reflecting gradients in oxygen availability, nutrient concentrations and organic matter quality (Andersen et al., 2013). Distinct archaeal, bacterial and methane-cycling communities occupy different ecological niches within the peat profile. Too et al. (2018) showed that peat depth was one of the strongest determinants of bacterial community structure, while Dom et al. (2021) linked prokaryotic community composition directly to carbon biogeochemical cycling across tropical peat domes. Dhandapani et al. (2020b) found consistent change in microbial community structure across land-uses with depth going down to 2 m below surface.

Our understanding of ecosystem health has traditionally emphasized vegetation structure, hydrology and wildlife populations. Yet these observable characteristics are underpinned by microbe-mediated processes. Nutrient mineralisation supports tree growth especially in these nutrient poor conditions, microbial competition influences pathogen suppression and microbial metabolism regulate greenhouse gas fluxes. Therefore, maintaining healthy microbial communities is essential not only for peat functioning but for sustaining the broader ecological networks that depend upon peatland ecosystems (Andersen et al., 2013; Too et al., 2018).

## Land-use change as a driver of change in microbial composition

Large areas of tropical peatlands have been converted to agriculture; this is particularly relevant in Southeast Asia that recorded the greatest rate of deforestation in early 21^ st^ century (Hansen et al., 2013). Oil palm is the biggest driver of deforestation in Southeast Asia (Miettinen et al., 2012, 2016), which is also the most consumed vegetable oil and the fastest expanding equatorial crop in the world (Lam et al., 2019). Such land-use changes are accompanied by drainage, increased aeration and altered vegetation composition (Dhandapani et al., 2019a). These environmental changes substantially reshape microbial communities throughout the peat profile.

Agricultural conversion of tropical peatlands not only alters peat properties but also restructures microbial communities, promoting fungal populations capable of producing large quantities of N_2_O (Yanai et al., 2007). Because N_2_O is a potent greenhouse gas and ozone-depleting substance, microbial community shifts induced by land-use change can have consequences that extend from local soil health to global climate and planetary health (Yanai et al., 2007).

Considering oil palm already covers a large area of tropical peatlands, we need to focus on the wise use and management of oil palm on peat to reduce environmental consequences (Dhandapani et al., 2020a, 2024). Studies employing phospholipid fatty acid profiling demonstrate that microbial community structures differ significantly among intact forest, drained, burned and oil palm peatlands. Furthermore, microbial composition changes consistently with depth, highlighting the importance of considering vertical ecological connectivity rather than focusing solely on surface soils. Disturbance driven shifts include changes in phenotypic bacterial dominance patterns and altered functional composition, including changes in fungi to bacteria ratios and microbial stress indicators (Dhandapani et al., 2020b).

From a One Health perspective, these microbial reorganizations should not be viewed merely as ecological consequences of land-use change. Rather, they represent fundamental alterations to ecosystem functioning that potentially influence nutrient availability, plant productivity, biodiversity support and greenhouse gas dynamics. The resulting impacts extend beyond soil health and propagate throughout ecological and societal systems. In fact, Kwon et al. (2013) provides mechanistic evidence that anthropogenic peatland drainage alters microbial community structure and function in ways that amplify carbon losses during drought events. As climate change is expected to increase drought frequency in many tropical regions, degraded peatlands may become disproportionately important sources of greenhouse gases, creating positive feedback between ecosystem degradation and global climate change. Furthermore, Kamal et al. (2009) provides evidence that tropical peatland microorganisms represent an underexplored resource for drug discovery. The loss of peatland biodiversity through drainage, fire and land conversion could therefore result not only in ecological degradation and carbon emissions, but also in the loss of microbial genetic resources with potential benefits for human health.

A useful conceptual shift is therefore to regard land-use change as a form of microbial ecosystem engineering, whereby anthropogenic modification of environmental conditions restructures microbial communities, subsequently affecting the services they provide.

## Hydrology: the master regulator of peatlands that connect microbiomes and One Health outcomes

Across virtually all tropical peatland studies, unsurprisingly hydrology emerges as the dominant environmental regulator. Water-table depth influences oxygen availability, redox conditions, nutrient transformations and organic matter decomposition. Consequently, it governs microbial community structure and functional potential throughout the peat profile (Andersen et al., 2013; Dhandapani et al., 2020b).

Fire and drainage provide compelling examples. Tropical peat fires can extend deep belowground, fundamentally modifying peat physical and chemical properties (Turetsky et al., 2015; Dhandapani and Evers, 2020). Recent work demonstrates that post-fire increases in methane emissions are associated with depth-dependent changes in microbiome composition, including enrichment of methanogenic microorganisms and reductions in methane-oxidizing taxa (Bandla et al., 2024). These shifts are linked to altered hydrological conditions and changes in aboveground vegetation.

Microbial studies consistently demonstrate strong depth-dependent patterns. Both Jackson et al. (2009), Azizan et al. (2024) observed distinct microbial communities between surface and deeper peat layers, reflecting changing anaerobic conditions and substrate availability. Specific microbial groups associated with carbon and sulfur cycling become increasingly abundant in deeper anaerobic peat.

This evidence supports a simple conceptual pathway that Hydrological disturbance alters biogeochemical conditions that effects Microbial composition, which then results in altered biogeochemical cycling, finally resulting in ecosystem service disruption resulting in devastating impacts on One Health. Under this framework, water table management becomes more than a hydrological intervention. It becomes an approach to managing microbiomes and the ecosystem services they regulate.

## Linking peat, plant, animal, and human health

The greatest opportunity for advancing One Health lies in explicitly connecting peatland microbiomes with plant, animal and human health outcomes.

Healthy peatland vegetation depends upon microbial regulation of nutrient acquisition, decomposition and organic matter turnover. In nutrient-poor peat soils, microbial communities play a vital role in maintaining plant productivity and supporting forest regeneration (Turjaman et al., 2006; Too et al., 2018). Altered microbial communities may therefore influence restoration success and ecosystem recovery trajectories.

Wildlife populations depend upon habitats supported by functioning peatland ecosystems (Yule, 2010). Changes in microbiome-mediated nutrient cycling can influence vegetation composition, food web dynamics and habitat quality. Although direct links between peatland microbiomes and wildlife health remain insufficiently studied, there is increasing recognition that ecosystem degradation can weaken ecological resilience across trophic levels.

Human health connections are perhaps the most visible. Peatland degradation contributes to severe fire and haze events that affect respiratory health across Southeast Asia (Tan et al., 2009; Page and Hooijer, 2016). Degradation also compromises water regulation, flood prevention, climate stability and ecosystem services upon which local livelihoods depend, where the acute impacts can be catastrophic. Because microbial processes strongly influence both decomposition and greenhouse gas production, microbial communities indirectly affect environmental conditions with significant public health implications. In addition, extraction of novel anticancer compounds, and antibiotics have much more direct medical implications for human health (Kamal et al., 2009; Goh et al., 2020)

Collectively, these interactions support a broader view of peatland microbiomes as mediators of planetary health rather than solely drivers of belowground ecosystem processes.

## Toward microbiome-informed restoration

Peatland restoration programmes commonly evaluate outcomes using hydrological indicators, vegetation recovery and greenhouse gas emissions. Although these metrics provide valuable information, they may overlook critical components of ecosystem recovery. Evidence suggests that microbial communities remain sensitive to disturbance and exhibit depth-dependent responses to environmental change (Dhandapani et al., 2020b). Consequently, restoration success should also be evaluated through recovery of microbial ecosystem functions and community stability.

This perspective supports development of a microbiome-informed restoration framework incorporating indicators such as, microbial community composition, microbial biomass, methanogen-to-methanotroph ratios, fungi to bacteria ratios, depth level microbial interactions with restoration, aboveground vegetation and peat microbe interactions etc. Such indicators could complement existing restoration metrics and provide mechanistic insights into ecosystem resilience. Ultimately, successful restoration should aim not merely to re-establish vegetation cover but to re-establish ecological processes supported by functional microbial communities.

The increasing urgency of climate change mitigation and ecosystem restoration has stimulated interest in novel interventions designed to accelerate peatland recovery and reduce greenhouse gas emissions. These include biochar amendments, microbial inoculants, paludiculture systems, engineered rewetting approaches and carbon-enhancement technologies. While many of these interventions show potential promise for improving peatland function, their effects on indigenous microbial communities remain insufficiently understood.

## Novel technologies and interventions: biochar as a case study

Among emerging technologies, biochar is widely regarded as a suitable amendment for low-quality acidic soils because it can improve nutrient and water retention while acting as a stable carbon sink (Schmidt et al., 2021). This is especially relevant for degraded or ‘wasted’ peatlands from prolonged oil palm cultivation, that start exhibiting mineral soil like characteristics (Dhandapani et al., 2019a), where key ecosystem services such as carbon sequestration and water storage are diminished, increasing risks of flooding and climate change. Furthermore, oil palm plantation produce large quantities of biomass (Kong et al., 2014), and converting them to biochar could provide an alternative to current residue management practices, where biomass is either burned on-site, used as fuel locally (field waste) or in export markets (mill waste), or left in the field where it may contribute to Ganoderma disease problems (Periasamy et al., 2026).

However, evidence regarding biochar effects on tropical peatland microbiomes remains extremely limited. Most current understanding is extrapolated from agricultural mineral soils and may not apply to tropical peatlands, where ecosystem functioning is tightly regulated by highly specialized microbial communities adapted to acidic, waterlogged and nutrient-limited conditions (Andersen et al., 2013). This Perspective argues that biochar should not be evaluated through just surface level processes, but through its influence on microbial indicators across the peat depth profile, as discussed in the previous section. Attention should also be given to the pathogen-suppression potential of biochar, especially against Ganoderma, a major constraint in oil palm cultivation (Judijanto, 2026). Such an approach would align restoration science with One Health principles by recognizing the role of peat microorganisms in ecosystem functions and health.

## Policy implications and conclusions: from ecological indicators to natural capital assets

Current peatland governance largely focuses on carbon emissions, water management and biodiversity conservation. While these priorities remain essential, microbial communities are rarely incorporated into policy frameworks despite underpinning many targeted outcomes. Peatland microbiomes should be recognized as a form of natural capital. This recognition would have implications for several policy domains. First, restoration guidelines could include microbiome recovery targets alongside hydrological and vegetation indicators. Second, carbon accounting and nature-based climate solutions could integrate microbial functional indicators to improve predictions of greenhouse gas dynamics and restoration effectiveness. Third, national and global One Health strategies could expand environmental health considerations to include soil microbial health. At an international level, microbial indicators could contribute to reporting frameworks associated with biodiversity conservation, wetland protection and peatland restoration initiatives. Such an approach would acknowledge that ecosystem services emerge from biological processes that are frequently invisible within conventional environmental monitoring systems.

Tropical peatland microbiomes represent a missing link in contemporary One Health discourse (Figure 1). By regulating carbon cycling, nutrient turnover, ecosystem resilience and greenhouse gas production, microorganisms connect soil processes with plant productivity, wildlife habitat quality, human wellbeing and climate regulation. Emerging evidence demonstrates that land-use change, drainage and fire fundamentally reorganize microbial communities, potentially altering the delivery of ecosystem services. Therefore, microbiomes should be regarded not simply as indicators of peatland condition but as foundational components of ecosystem health and natural capital. Incorporating microbial perspectives into restoration, monitoring and policy frameworks offers an opportunity to improve ecosystem management while advancing One Health objectives.

**FIGURE 1:**
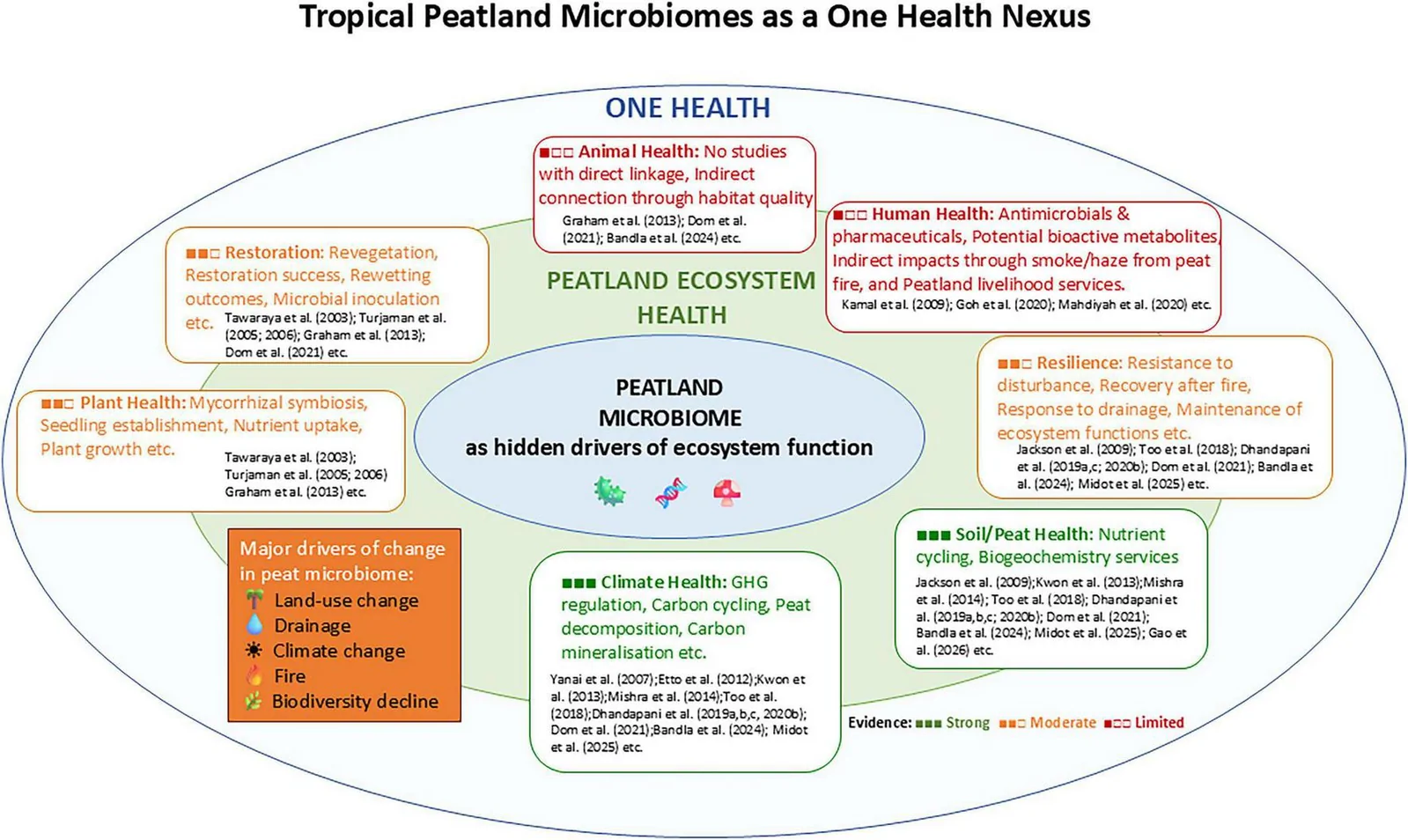
A brief graphical representation of the current understanding of tropical peatland microbes as a One Health nexus.

Within a One Health framework, tropical peatland microbiomes provide a unique and highly impactful model system for investigating how belowground biodiversity influences ecosystem functioning, plant health, wildlife habitat quality, climate regulation and ultimately human wellbeing. Their conservation may therefore be as important as the protection of aboveground biodiversity in maintaining ecosystem services and safeguarding planetary health.

## Data Availability

The original contributions presented in this study are included in this article/supplementary material, further inquiries can be directed to the corresponding author.
